# The Determination of Step Frequency in 3-min Incremental Step-in-Place Tests for Predicting Maximal Oxygen Uptake from Heart Rate Response in Taiwanese Adults

**DOI:** 10.3390/ijerph19010563

**Published:** 2022-01-05

**Authors:** Fang Li, Chun-Hao Chang, Chia-An Ho, Cheng-You Wu, Hung-Chih Yeh, Yuan-Shuo Chan, Jia-Yu Cheng, Wen-Sheng ChangChien, Chin-Shan Ho

**Affiliations:** 1 Graduate Institute of Sports Science, National Taiwan Sport University, Taoyuan 333325, Taiwan; lif848332@gmail.com (F.L.); hao781106@ntsu.edu.tw (C.-H.C.); 1100207@ntsu.edu.tw (C.-Y.W.); yeh19970305@gmail.com (H.-C.Y.); 1080219@ntsu.edu.tw (J.-Y.C.); 2 Department of Sport Promotion, National Taiwan Sport University, Taoyuan 333325, Taiwan; 1075032@ntsu.edu.tw; 3 Department of Special Education, National Taipei University of Education, Taipei 10671, Taiwan; yschan@tea.ntue.edu.tw; 4 Innovation Lab., H2U Corporation, New Taipei 23143, Taiwan; changchien.ws@gmail.com

**Keywords:** maximal oxygen uptake, 3-min incremental step-in-place, step frequency, multiple regression model

## Abstract

The maximal oxygen uptake (VO_2max_) prediction models established by step tests are often used for evaluating cardiorespiratory fitness (CRF). However, it is unclear which type of stepping frequency sequence is more suitable for the public to assess the CRF. Therefore, the main purpose of this study was to test the effectiveness of two 3-min incremental step-in-place (3MISP) tests (i.e., 3MISP_30s_ and 3MISP_60s_) with the same total number of steps but different step-frequency sequences in predicting VO_2max_. In this cross-sectional study, a total of 200 healthy adults in Taiwan completed 3MISP_30s_ and 3MISP_60s_ tests, as well as cardiopulmonary exercise testing. The 3MISP_30s_ and 3MISP_60s_ models were established through multiple stepwise regression analysis by gender, age, percent body fat, and 3MISP-heart rate. The statistical analysis included Pearson’s correlations, the standard errors of estimate, the predicted residual error sum of squares, and the Bland–Altman plot to compare the measured VO_2max_ values and those estimated. The results of the study showed that the exercise intensity of the 3MISP_30s_ test was higher than that of the 3MISP_60s_ test (% heart rate reserve (HRR) during 3MISP_30s_ vs. %HRR during 3MISP_60s_ = 81.00% vs. 76.81%, *p* < 0.001). Both the 3MISP_30s_ model and the 3MISP_60s_ model explained 64.4% of VO_2max_, and the standard errors of the estimates were 4.2043 and 4.2090 mL·kg^−1^·min^−1^, respectively. The cross-validation results also indicated that the measured VO_2max_ values and those predicted by the 3MISP_30s_ and 3MISP_60s_ models were highly correlated (3MISP_30s_ model: r = 0.804, 3MISP_60s_ model: r = 0.807, both *p* < 0.001). There was no significant difference between the measured VO_2max_ values and those predicted by the 3MISP_30s_ and 3MISP_60s_ models in the testing group (*p* > 0.05). The results of the study showed that when the 3MISP_60s_ test was used, the exercise intensity was significantly reduced, but the predictive effectiveness of VO_2max_ did not change. We concluded that the 3MISP_60s_ test was physiologically less stressful than the 3MISP_30s_ test, and it could be a better choice for CRF evaluation.

## 1. Introduction

According to a report from the World Health Organization (WHO), cardiovascular disease is the world’s leading cause of death. The number of deaths from ischemic heart disease account for 16% of the total deaths in the world, and strokes account for 11% of the total deaths [1]. Cardiorespiratory fitness (CRF) is an important indicator of cardiovascular health [2,3,4]. Through CRF assessment, current and future health conditions can be objectively predicted. Generally speaking, a higher CRF level indicates a lower risk of cardiovascular disease, all-cause mortality, and medical costs in later life [5,6,7]. Therefore, the evaluation of CRF is very important, even in healthy adults. The most accurate CRF assessment method for adults is the graded cardiopulmonary exercise testing (CPET) until volitional exhaustion on a treadmill or cycle ergometer. Oxygen (O_2_) consumption is the product of cardiac output and arteriovenous O_2_ difference (a–vO_2_ diff) [8,9,10]. Cardiac output is strongly related to fat-free body mass, which is higher in overweight than normal-weight adults [11]. The a–vO_2_ diff decreases with advancing age [8]. During exercise, with the increase of intensity, the skeletal muscles need to extract more O_2_ from the blood, thus resulting in the increase of a–vO_2_ diff. The plateau in O_2_ consumption during CPET represents the maximum upper limit of CRF [12]. Accordingly, the maximal oxygen uptake (VO_2max_) obtained with the gas analyzer is considered by the WHO as the best indicator for evaluating CRF. The American College of Sports Medicine’s guidelines for exercise testing and prescription classifies the age-and gender-associated CRF based on VO_2max_ as very poor, poor, fair, good, excellent, and superior [7], indicating a decline in CRF across the lifespan. The VO_2max_ of men is significantly higher than that of women; the main reason for this gender-related difference is physiologic differences, including body composition, heart size, and lung volume [13,14,15]. The direct method of measuring VO_2max_ depends on the availability of laboratory equipment and the professional operation of said equipment by well-trained staff. For the direct measurement of VO_2max_, healthy individuals should perform the maximum stress test; it does not pose a risk for health under the management of the professional, while low-intensity tests are recommended for individuals with health problems or physical disabilities [4,7]. For these reasons, it cannot be carried out universally. Therefore, it is necessary to develop a low-risk, low-cost, and more convenient indirect measurement program for VO_2max_ to assess CRF levels.

The submaximal exercise-based prediction models to estimate VO_2max_ have moderate to high levels of accuracy [16], which can explain individual-specific exercise responses and identify the longitudinal changes in CRF [17]. Many scholars in the past have explored submaximal exercise protocols. Common test methods include the 6-min walk test [18], the 20-m shuttle run (20MSR) test [19], and step-up tests [20,21]. Those scholars used exercise parameters such as heart rate, speed, and distance as predictors of VO_2max_ and combined age, gender, and physiological characteristics to establish a VO_2max_ prediction formula to evaluate CRF levels. Among them, the 6-min walk test (6MWT) is often used to assess the CRF level of older adults and clinical patients [13,18]. The American Thoracic Society has published 6-min walk test procedures and instructions for interpretation [22], requiring individuals to walk back and forth along a 30-m walkway within 6 min [23]. The CRF could be differentiated as very low, low, excellent, or superior according to the 6MWT distance, but which had no ability to identify adults with regular and good CRF [13]. The 20MSR is a field test widely used to evaluate CRF [19]. This test is simple and easy to manage, requires less equipment, and can be used to test by multiple people at the same time, but it requires a 20-m testing space. Although the accuracy of VO_2max_ prediction by submaximal exercise tests is lower than that of maximal exercise tests, submaximal exercise tests have a lower risk of accidents and require less time and equipment. When VO_2max_ cannot be measured directly due to considerations such as safety, the environment, equipment, and personnel training, submaximal exercise can be used to measure VO_2max_ indirectly to facilitate the identification and follow-up of adult CRF.

Step-up tests are one of the most widely used submaximal exercise tests for predicting VO_2max_. Compared with other submaximal exercise tests, this test has advantages in terms of time, space, and cost, and it can be performed indoors or outdoors. Step-up tests mainly include the Astrand-Ryhming [24], Young Men’s Christian Association [20,21], Queen’s College [25], and Harvard Step [26] tests. Different step-up test protocols have different stepping frequencies, test durations, and numbers of test phases, but the main purpose is to evaluate CRF [20]. These tests require participants to continuously step onto and off a box with a fixed height (20–50 cm) at a stepping frequency of 22–30 steps/min for 3–5 min, and the CRF level is finally evaluated according to the heart rate during or after exercise [7,24,27]. This stepping test requires high balance, coordination, and muscle strength of the lower extremities. It is not suitable for individuals with poor physical fitness or balance problems; extreme obesity; and lower extremity injuries [7,28,29]. Therefore, the choice of a submaximal exercise protocol for predicting VO_2max_ must meet the physical fitness levels of a variety of adults.

Recent cross-sectional studies have proposed another step test method, namely, the 3-min incremental step-in-place (3MISP) test, for predicting the VO_2max_ of healthy adults, and the results have been significant [30,31]. The 3MISP test movement is simple, requires less space and equipment, has a short duration, and does not require the professional operation of equipment. According to individual differences, the test uses the middle of the line between the anterior superior iliac spine and the midpoint of the patella as the target of knee elevation during stepping, and it requires no step-up box, so the safety index is higher than that of the step-up tests [31]. The VO_2max_ prediction model established by 3MISP can improve the accuracy of VO_2max_ prediction, and it has the advantages of indoor testing, convenient management, simplicity, and ease of use [31]. It can also be used for CRF tests of ordinary people in the homes, at work, or elsewhere. However, it is still unclear which type of 3MISP stepping frequency sequence is more suitable for CRF measurement by the general public. Different stepping frequencies may result in different physiological responses in participants. In the past, few studies have analyzed the effects of different stepping frequency sequences on individual physiological responses and the effectiveness of predicting VO_2max_. Therefore, the purpose of this cross-sectional study was to evaluate the impact of two 3MISP tests with different step-frequency sequences (i.e., 3MISP_30s_ and 3MISP_60s_ tests) on the individual’s physiological responses and the effectiveness of VO_2max_ estimation. In this study, we assumed that under the same total number of steps, the 3MISP_60s_, with fewer changes in stepping frequency, would cause less physiological stress on participants and have a lower exercise intensity, and that the effectiveness of VO_2max_ estimation would not be inferior to that of the 3MISP_30s_ test, which has more changes in stepping frequency.

## 2. Materials and Methods

### 2.1. Study Design

This cross-sectional study was approved by the Institutional Review Board (IRB) of the Industrial Technology Research Institute (IRB No: IRB-APP-F02-106-009). The study protocol included two 3MISP tests (3MISP_30s_ and 3MISP_60s_) at a given step count (360 steps) with different frequency sequences, and a graded CPET. In this study, a Polar H10 Heart Rate Monitor (Polar Electro Oy, Finland) was used to continuously monitor the heart rate responses of the participants during the CPET and 3MISP tests. The VO_2max_ of the participants was directly measured by CPET to exhaustion on an electromagnetic bicycle ergometer (Excalibur Sport Ergometer, Lode BV, The Netherlands) and cardiopulmonary exercise testing system (Vmax Encore 29 System, VIASYS Healthcare Inc., Yorba Linda, CA, USA). Because there is a significant correlation between the directly measured VO_2max_ value and the heart rate change during the 3MISP tests [30,31], this study used 3MISP-heart rate (HR) as a predictor of VO_2max_ and combined age, gender, and physical characteristics to establish two VO_2max_ prediction models (the 3MISP_30s_ and 3MISP_60s_ models). To evaluate the reliability and validity of these two VO_2max_ prediction models, this study used the predicted residual error sum of squares (PRESS) cross-validation procedure to verify the models.

### 2.2. Subjects

In this cross-sectional study, 140 adults in Taiwan (i.e., the training group: 70 men, 70 women) were used for developing a VO_2max_ prediction model and another 60 Taiwanese adults (i.e., the testing group: 30 men, 30 women) were used for cross-verifying the prediction model. Table 1 shows the basic characteristics of the subjects in the training and testing groups. A total of 100 healthy men and 100 healthy women completed the test. Prior to the implementation of the study, all subjects completed the informed consent forms. In this study, the body mass index (BMI) was calculated by dividing the weight (kg) by the square of the height (meters). The participants’ body weight and percent body fat (PBF) were measured by InBody^®^ 570 Body Composition Analyzer (Biospace, Inc., Seoul, Korea) [32]. All participants in this study were randomly and openly recruited, through social networks and posting advertisements in public spaces. Inclusion criteria were healthy adults aged 20–64 years, living in Taiwan. Pregnant women; individuals with lung, cardiovascular, or metabolic diseases; and those with neurological, muscular, or skeletal diseases were excluded.

### 2.3. 3-min Incremental Step-In-Place Tests

**Figure 1:**
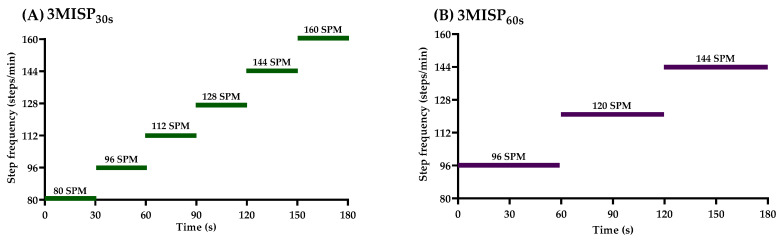
The step frequencies of the 3MISP_30s_ (**A**) and 3MISP_60s_ (**B**) tests.

In this study, all participants performed two 3MISP tests (i.e., the 3MISP_30s_ and 3MISP_60s_ tests) with the same total number of steps (360 steps) and different step-frequency sequences (Figure 1). The 3MISP_30s_ test started at 80 SPM and then increased by 16 SPM every 30 s (Figure 1A), in which there were 6 stages of step frequency. The 3MISP_60s_ test started at 96 SPM and then increased by 24 SPM every 60 s (Figure 1B), in which there were 3 stages of step frequency. Before the 3MISP test, participants were asked to wear a chest strap-style Polar H10 heart rate monitor to continuously monitor their heart rate responses during stepping. Once the heart rate monitor was worn, the middle of the line connecting the participant’s anterior superior iliac spine and the midpoint of the patella was measured as the target of their knee elevation when they stepped. After the start of the 3MISP test, participants needed to follow the rhythm of the metronome to raise their knees and step for 3 min while ensuring that the knees were raised to the required height each time. If the participant could not keep up with the rhythm or reach the required knee lift height for 30 s, the test was terminated and removed from the data analysis. In addition, for safety reasons, participants continued stepping at 80 SPM for 30 s after the 3MISP test and then completed 30 s of standing rest. The heart rates of each participant at the beginning (HR0) of the 3MISP exercise, as well as at the 1st (HR1), 2nd (HR2), and 3rd (HR3) minutes of exercise, and at the 1st minute (HR4) after the end of the 3MISP test, were recorded for subsequent data analysis.

### 2.4. Cardiopulmonary Exercise Testing

In this study, the VO_2max_ of all participants was measured directly on the electromagnetic bicycle ergometer with graded CPET until volitional exhaustion and with the cardiopulmonary exercise testing system. During the CPET process, participants wore a Polar H10 heart rate monitor and a suitable air-gathering mask (Hans-Rudolph), and the Borg Rating of Perceived Exertion (RPE, 6–20 scale) was used to evaluate the participants’ degree of fatigue during the test. The heart rate monitor was mainly used to monitor the heart rate responses of the participants during the CPET process. The breath-by-breath air volume and the contents of O_2_ and CO_2_ were measured through the sampling line connected to the mask and the digital flow sensor. The initial load of CPET was 25 W, and then the resistance was increased by 15 W every 2 min until the participant could no longer maintain the pedaling rate of 70 revolutions per minute. In this study, the criteria for VO_2max_ was the participant meeting three of the following conditions: a respiratory ratio (CO_2_/O_2_) ≥ 1.10, a maximum heart rate of more than 90% of the age-predicted maximal HR (220—age), and an RPE > 17; even if the exercise load was increased, the oxygen uptake still tended to be stable [7,31,33].

### 2.5. Statistical Analysis

In this study, the statistical software SPSS (version 20, IBM Corp., New York, NY, USA) was used for statistical analysis. All values are presented in the form of means ± standard deviations. The significance level was set to *p* < 0.05. The normality assumption was checked using Shapiro–Wilk test. The independent samples t-test (normally distributed data) and Mann–Whitney U test (non-normally distributed data) were used to compare the physical characteristics and heart rate responses between the training and testing groups. Cohen’s *d* effect sizes were calculated to compare the practical difference between groups, where the effect sizes <0.2, 0.2~0.5, 0.5~0.8, and >0.8 were indictive of a trivial, small, moderate, and large difference, respectively [34]. Pearson’s correlation coefficients were used to analyze the linear relationship between the actual measured value of VO_2max_ and gender, age, PBF, and 3MISP-HR in the training group, and the validity of the VO_2max_ prediction model. The correlation coefficients were interpreted by Schober et al. (2018), with 0.90~1.00 categorized as very strong, 0.70~0.89 as strong, 0.40~0.69 as moderate, and 0.10~0.39 as weak correlation [35]. Through multiple stepwise regression analysis, two VO_2max_ prediction models (i.e., the 3MISP_30s_ and 3MISP_60s_ models) were established by five parameters: gender, age, PBF, HR0, and ΔHR3−HR4. The multiple coefficients of determination (R^2^), the absolute standard errors of estimate (SEE), and relative SEE (SEE%) were used to evaluate and compare the accuracies of the two VO_2max_ prediction models. The PRESS statistical method was used to cross-validate the VO_2max_ prediction model [31,36]. The PRESS R^2^ (R^2^*_p_*) is defined as 1—(PRESS/SS_total_), while the PRESS SEE (SEE*_p_*) is calculated as $\sqrt{\mathrm{RRESS}/N}\;$ [37]. Bland–Altman plots were used to compare the differences between the actual measured and estimated values of VO_2max_, which allowed us to visualize the data distribution around the line of zero [17,23,38].

## 3. Results

**Figure 2:**
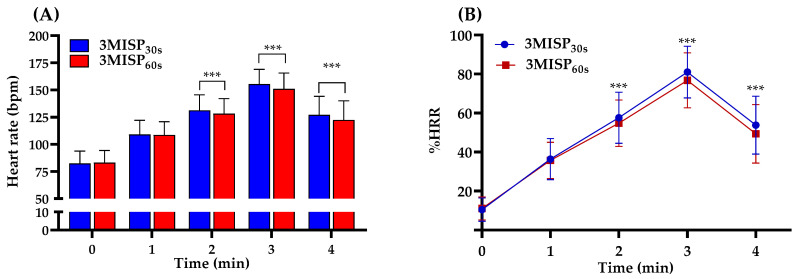
(**A**) Participants’ heart rate responses during the 3MISP_30s_ and 3MISP_60s_ tests (N = 200); (**B**) %HRR (the percentage of heart rate reserve) curves under the 3MISP_30s_ and 3MISP_60s_ tests; *** Significant difference (*p* < 0.001) in heart rate (A) or %HRR (B) between the 3MISP_30s_ and 3MISP_60s_ tests.

Table 2 lists the heart rate responses of the training group and the testing group during the 3MISP_30s_ and 3MISP_60s_ tests. There was no significant difference in the heart rate responses during the 3MISP_30s_ or 3MISP_60s_ test between the two groups (*p* > 0.05). Figure 2 shows the heart rate responses of all participants in these two 3MISP tests, and their corresponding exercise intensities. The results of the study showed that the heart rate of the participants at the third minute during the 3MISP_30s_ test was significantly higher than the heart rate at the third minute during the 3MISP_60s_ test (3MISP_30s_: 156 ± 14 bpm, 3MISP_60s_: 151 ± 15 bpm, *p* < 0.001). The exercise intensity tested by 3MISP_30s_ was higher than the exercise intensity tested by 3MISP_60s_ (%heart rate reserve (HRR) during 3MISP_30s_ vs. %HRR during 3MISP_60s_ = 81.00% vs. 76.81%, *p* < 0.001).

**Figure 3:**
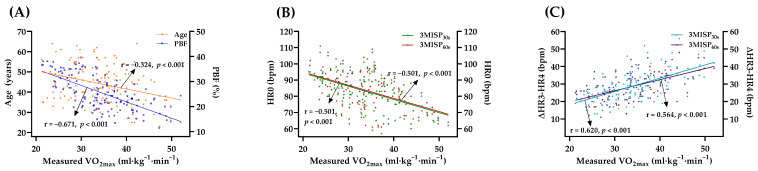
Pearson’s correlation coefficients for the correlations between VO_2max_ and age, PBF (**A**), HR0 (**B**), and ΔHR3–HR4 (**C**) in the training group (N = 140), showing the regression line. HR0, heart rate at the beginning of the 3MISP test; ΔHR3−HR4, the difference between the heart rate at the 3rd minute during the exercise and the heart rate at the 1st minute after the end of the 3MISP test.

Figure 3 shows the correlations between the actual measured VO_2max_ and age, PBF, and 3MISP-HR in the training group. Pearson’s correlation coefficients showed a positive correlation between gender (women = 0, men = 1) and VO_2max_ (r = 0.453, *p* < 0.001). There was a negative correlation between VO_2max_ and age or PBF (age: r = −0.324, PBF: r = −0.671, both *p* < 0.001). There was a negative correlation between HR0 and VO_2max_ (3MISP_30s_: r = −0.501, 3MISP_60s_: r = −0.501, both *p* < 0.001). There was a positive correlation between ΔHR3−HR4 and VO_2max_ (3MISP_30s_: r = 0.620, 3MISP_60s_: r = 0.564, both *p* < 0.001).

Two multiple regression models (i.e., the 3MISP_30s_ and 3MISP_60s_ models) for predicting VO_2max_ using age, gender, PBF, HR0, and ∆HR3−HR4 are presented in Table 3, as well as the results of cross-validation. All variables in the 3MISP_30s_ and 3MISP_60s_ models were independently related to VO_2max_. In this study, the two VO_2max_ prediction models showed similar multiple correlations and SEE (3MISP_30s_ model: R^2^ = 0.644, SEE = 4.2043 mL·kg^−1^·min^−1^; 3MISP_60s_ model: R^2^ = 0.644, SEE = 4.2090 mL·kg^−1^·min^−1^). The cross-validation result of the PRESS statistical method showed that the changes in R^2^ and SEE of the 3MISP_30s_ and 3MISP_60s_ models were very small (∆R^2^ ≤ 0.007, ∆SEE ≤ 0.075 mL·kg^−1^·min^−1^).

**Figure 4:**
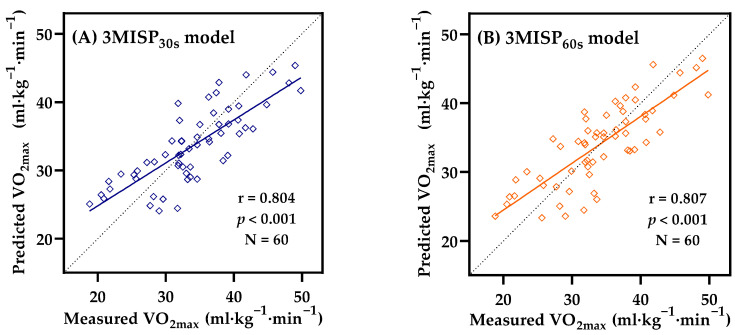
Pearson’s correlations between the measured VO_2max_ values and those predicted by the 3MISP_30s_ (**A**) and 3MISP_60s_ (**B**) models in the testing group (N = 60).

Figure 4 shows the Pearson’s correlations in the testing group between the measured VO_2max_ values and those predicted by the 3MISP_30s_ and 3MISP_60s_ models. The results of this study showed that the VO_2max_ values estimated by both models were highly correlated with the actual measured values of VO_2max_ (3MISP_30s_: r = 0.804, 95% confidence interval (CI) = 0.715-0.872; 3MISP_60s_ model: r = 0.807, 95% CI = 0.720-0.875; both *p* < 0.001). The prediction values (R^2^) from the 3MISP_30s_ and 3MISP_60s_ models were moderate, and the prediction errors were relatively large (3MISP_30s_: R^2^*_p_* = 0.646, SEE*_p_* = 4.180 mL·kg^−1^·min^−1^, SEE*_p_*% = 12.31%; 3MISP_60s_: R^2^*_p_* = 0.651, SEE *_p_* = 4.134 mL·kg^−1^·min^−1^, and SEE*_p_*% = 12.18%).

**Figure 5:**
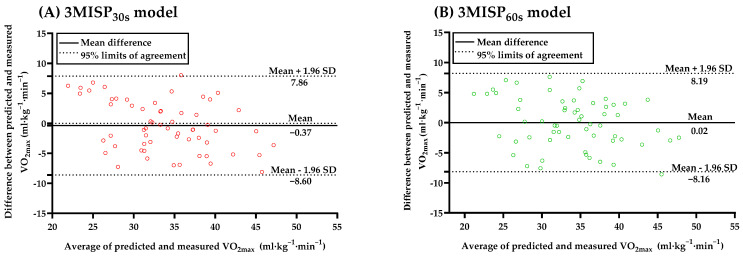
The differences between the predicted VO_2max_ values and those measured by the 3MISP_30s_ (**A**) and 3MISP_60s_ (**B**) models in the testing group (N = 60) in Bland–Altman Plots.

Figure 5 shows the differences between the predicted VO_2max_ values and those measured by the 3MISP_30s_ and 3MISP_60s_ models with the 95% limits of agreement (LoAs) in Bland–Altman Plots. The average differences between the measured VO_2max_ values and those predicted by the 3MISP_30s_ model (LoAs = −8.60 to 7.86 mL·kg^−1^·min^−1^) and 3MISP_60s_ model (LoAs = −8.16 to 8.19 mL·kg^−1^·min^−1^) were −0.37 and 0.02 mL·kg^−1^·min^−1^, respectively, and none of them reached the level of significance (*p* > 0.05). However, the 3MISP_30s_ and 3MISP_60s_ models seem to underestimate the real VO_2max_ of participants with high VO_2max_ values, whereas they overestimate it for participants with low VO_2max_ values.

## 4. Discussion

CRF is one of the basic elements of healthy physical fitness. For the assessment of large-scale CRF, the subjects have a large age span and different physical conditions. To determine which step frequency sequence is more appropriate for healthy adults to use in CRF evaluation, this cross-sectional study compared the heart rate responses in 3MISP_30s_ and 3MISP_60s_ tests and the accuracies of VO_2max_ prediction models. The results of this research show that the 3MISP_60s_ test, which has fewer step-frequency changes, significantly reduces the exercise intensity while maintaining the same total number of steps (work amount). The subject’s heart rate responses in the 3MISP_60s_ test are lower than those in the 3MISP_30__s_ test. We also confirmed that the predictive effectiveness of VO_2max_ did not decrease when the exercise intensity was reduced. Therefore, a simple, safe, and effective 3MISP_60s_ test program may be a better choice as a field test for evaluating CRF across age groups.

During the progressive stepping test, the ability of the subject to perform incremental exercise loads, and the physiological response to exercise loads, can be observed [39]. The heart rate is an important physiological indicator of the function of the heart and the circulatory system. The heart rate, measured continuously, can simply, objectively, and directly reflect exercise intensity and the individual’s adaptability to the exercise load, without the requirement of familiarization trials [3,16]. Therefore, this study analyzed the individual heart rate responses during the 3MISP_30s_ and 3MISP_60s_ tests using continuous heart monitoring at different step-frequency sequences. The results of this study show that under the condition of the same total number of steps, the 3MISP_60s_ test, with fewer step-frequency changes, significantly reduces the exercise intensity at the second and third minutes (Figure 2B). The heart rate changes (HR0 and ΔHR3−HR4) during the 3MISP_30s_ and 3MISP_60s_ tests were all significantly correlated with the actual measured VO_2max_ (Figure 3B,C). This is consistent with the results of other studies using the step-test scheme to predict VO_2max_ and suggests that the CRF test, the subject’s basal heart rate, recovery heart rate, and heart rate during exercise are all negatively correlated with measured VO_2max_ [21,29,30,36,40]. Obviously, the change of heart rate during the 3MISP_30s_ and 3MISP_60s_ tests is one of the important factors for estimating VO_2max_. In the case of maintaining the same total number of steps, decreasing the times of 3MISP step-frequency changes can reduce the exercise intensity as well as improve safety. Because the subjects bear relatively low physiological load during the 3MISP_60s_ test, it may help to encourage more people to complete the CRF test. The relatively low physiological load can also increase the completion of the test movements and improve the effectiveness of the VO_2max_ prediction formula.

In this study, age, gender, and PBF were significantly correlated with measured VO_2max_ (Figure 3A), which is consistent with previous research results. In the past, many studies have shown that age, gender, and physical characteristics (BMI/PBF/waist circumference (WC)) are important factors in estimating VO_2max_. The addition of characteristic variables to the VO_2max_ prediction models led to decreased error, which may have been caused by the improvement of individual’s characteristics in personalization [41]. The PBF’s prediction of VO_2max_ is more accurate than those of BMI and WC, and it is a key factor for estimating VO_2max_ [21,30,36,42,43]. Therefore, in this study, age, gender, and PBF were used to establish the VO_2max_ estimation formula. In addition, heart rate is one of the variables most commonly used to develop the predictive models [16,41]. To further improve the accuracy of VO_2max_ estimation, this cross-sectional study also used the heart rate changes during the 3MISP_30s_ and 3MISP_60s_ tests as the predictive variable to establish the VO_2max_ estimation formula. The two VO_2max_ estimated models (i.e., the 3MISP_30s_ and 3MISP_60s_ models) based on age, gender, PBF, HR0, and ΔHR3−HR4 can explain 64.4% of the measured VO_2max_. The SEE values of the two models were also very close (Table 3). This shows that the 3MISP_60s_ test, despite its lower exercise intensity, is similar to the 3MISP_30s_ test. It can also predict the VO_2max_ of healthy adults very well (R^2^ = 0.644, SEE = 4.209 mL·kg^−1^·min^−1^). It can be seen that the prediction accuracy of VO_2max_ for submaximal exercises with lower intensity is not worse than that for submaximal exercises with higher exercise intensity.

In past cross-sectional studies, step-up tests have often been used to estimate adult VO_2max_ [20,21,29,36,39], with considerable results. According to age, gender, body weight, height, BMI, and heart rate during step-up tests, the R^2^ and SEE of the VO_2max_ estimation formula are 0.56–0.73 and 4.05–5.01 mL·kg^−1^·min^−1^, respectively. However, step-up tests usually require a step box with a height of 20–50 cm. Participants who are overweight or have knee injuries, abnormal gait, or impaired balance are likely to fall while stepping onto and off the box [30]. In the step-in-place test, the middle of the line between the anterior superior iliac spine and the midpoint of the patella is used as the target of knee elevation during stepping. No step-up box is required, so it is safer than the step-up test [30,31,44]. At present, the step-in-place test is mainly a part of the senior fitness test, which is used to assess the aerobic endurance of older adults (≥60 years). However, few related studies have examined the measurement of the CRF level of younger adults by the step-in-place test. To further improve the safety and universality of adult CRF examination, this study designed two 3MISP test methods (3MISP_30s_ and 3MISP_60s_) with different step-frequency sequences. Compared with previous studies that used the step-up test to predict VO_2max_, the R^2^ (0.644) and SEE (4.2043–4.2090 mL·kg^−1^·min^−1^) values of the two VO_2max_ prediction models established using 3MISP_30s_ and 3MISP_60s_ tests in this study are both acceptable (Table 3). They can predict the VO_2max_ of healthy adults relatively accurately.

The cross-validation results of this study showed that both the 3MISP_30s_ and 3MISP_60s_ models had a high level of cross-validity (Table 3). The measured VO_2max_ values and those predicted by the 3MISP_30s_ and 3MISP_60s_ models were highly correlated in the testing group (Figure 4). From Bland–Altman Plots, it was also found that the measured and estimated VO_2max_ obtained from the 3MISP_30s_ and 3MISP_60s_ models had high consistency (Figure 5). The accuracy of 3MISP-based predictions in this cross-sectional study was consistent with the previous research reporting that the VO_2max_ models were moderately to highly accurate [16]. There were significant relationships and agreements between the measured VO_2max_ and those estimated by submaximal exercise-based prediction models [16,17]. These results provided evidence for the effectiveness of the 3MISP_30s_ and 3MISP_60s_ models in predicting VO_2max_. Therefore, the two 3MISP tests with different step-frequency sequences proposed in this study, and the established VO_2max_ prediction models, are practicable. However, the exercise intensity of the 3MISP_60s_ test is relatively low, and the physiological load on the subjects is relatively small. Therefore, it may be a better choice to measure the adult CRF level in comparison with the 3MISP_30s_ test.

In summary, this study has three features. First, the two self-designed step-in-place test methods have the advantages of simplicity and ease of use. Second, under the condition of the same total number of steps (work amount), the 3MISP_60s_ test method, with fewer step-frequency changes, significantly reduces the exercise intensity and improves safety. Finally, it was successfully verified that the predictive effectiveness of VO_2max_ did not decrease when the exercise intensity was reduced.

There are several limitations in this cross-sectional study. A limitation of the study is that the mean age of all subjects was 43.72 ± 10.09 years, who are representative of healthy adults living in Taiwan. Thus, we could not verify whether the results may apply to older individuals, patients, or other racial groups. Another potential limitation is that our study established VO_2max_ prediction models using the cycle ergometer, a popular exercise mode, rather than the treadmill ergometry. Finally, although we have developed two 3MISP tests with different step-frequency sequences, the optimal step frequency is not known in this study, and further research will be needed to determine it.

## 5. Conclusions

The 3MISP_30s_ and 3MISP_60s_ tests are simple to operate, take up little time and space, and do not require expensive equipment or professionals to operate said equipment. This study has confirmed that the 3MISP tests of these two different step-frequency sequences can effectively predict the VO_2max_ of healthy adults. The 3MISP_60s_ test, despite its lower exercise intensity, is not inferior to the 3MISP_60s_ test in terms of the accuracy of predicting VO_2max_. Moreover, the 3MISP_60s_ test places a smaller physiological burden on the subjects than that of the 3MISP_30s_ test, and it is safer for CRF field tests in adult populations, and especially in older subjects. In this study, the 3MISP_60s_ test may be a better choice than the 3MISP_30s_ test for measuring the CRF level of healthy adults. However, a safer and more effective CRF test method for submaximal exercise is still to be pursued by researchers.

## Figures and Tables

**Table 1:** The basic characteristics of the subjects in the training and testing groups.

Characteristics	Training Group (N = 140)	Testing Group (N = 60)	Total (N = 200)	Effect Size
Age (years)	44.13 ± 9.63	42.77 ± 11.12	43.72 ± 10.09	0.13
Gender, N (%)				
Men	70 (50)	30 (50)	100 (50)	
Women	70 (50)	30 (50)	100 (50)	
Height (cm)	165.96 ± 7.84	166.48 ± 8.65	166.12 ± 8.07	−0.06
Body mass (kg)	67.30 ± 12.98	68.48 ± 13.29	67.65 ± 13.05	−0.09
PBF (%)	25.66 ± 6.77	26.64 ± 7.49	25.96 ± 6.99	−0.14
BMI (kg/m^2^)	24.24 ± 3.32	24.58 ± 3.47	24.34 ± 3.36	−0.10
VO_2max_ (mL·kg^−1^·min^−1^)				
Men	37.35 ± 6.60	36.85 ± 7.11	37.20 ± 6.72	0.07
Women	31.10 ± 5.76	31.06 ± 5.77	31.09 ± 5.74	0.01

Values are presented as the mean ± standard deviation. PBF, percent body fat; BMI, body mass index.

**Table 2:** Heart rate responses of the training group and testing group during the 3MISP_30s_ and 3MISP_60s_ tests.

	Training Group			Testing Group			*p*	Effect Size
	Total (N =140)	Women (N = 70)	Men (N = 70)	Total (N = 60)	Women (N = 30)	Men (N = 30)		
3MISP_30s_								
HR0 (bpm)	83 ± 11	84 ± 11	81 ± 12	82 ± 11	85 ± 11	79 ± 12	0.654	0.09
HR1 (bpm)	109 ± 13	113 ± 13	105 ± 11	109 ± 14	113 ± 15	104 ± 11	0.847	0.00
HR2 (bpm)	131 ±14	136 ± 15	127 ± 12	130 ± 15	136 ± 15	125 ± 13	0.649	0.07
HR3 (bpm)	155 ± 14	157 ± 15	152 ±13	157 ± 13	160 ± 12	154 ± 13	0.268	−0.15
HR4 (bpm)	126 ± 17	131 ± 18	121 ± 16	130 ± 16	134 ± 15	126 ± 16	0.088	−0.24
ΔHR3−HR4 (bpm)	29 ± 8	27 ± 8	31 ± 8	27 ± 9	26 ± 7	28 ± 10	0.154	0.23
3MISP_60s_								
HR0 (bpm)	83 ± 11	85 ± 11	82 ± 11	82 ± 11	85 ± 11	80 ± 11	0.614	0.09
HR1 (bpm)	109 ± 12	111 ± 13	106 ± 11	109 ± 13	113 ± 13	104 ± 11	0.926	0.00
HR2 (bpm)	128 ± 14	132 ± 15	125 ± 11	128 ± 14	132 ± 14	124 ± 13	0.884	0.00
HR3 (bpm)	150 ± 15	153 ± 16	148 ± 13	153 ± 14	156 ± 15	150 ± 14	0.255	−0.21
HR4 (bpm)	121 ± 18	125 ± 19	118 ± 15	125 ± 18	130 ± 19	120 ± 16	0.171	−0.22
ΔHR3−HR4 (bpm)	29 ± 8	28 ± 8	30 ± 7	28 ± 8	26 ± 8	31 ± 8	0.507	0.13

Values are presented as the mean ± standard deviation. HR0, heart rate at the beginning of the 3MISP test; HR1, heart rate at the first minute during the 3MISP test; HR2, heart rate at the second minute during the 3MISP test; HR3, heart rate at the third minute during the 3MISP test; HR4, heart rate at the first minute after the end of the 3MISP test; and ΔHR3−HR4, the difference between the heart rate at the 3rd minute during the exercise and the heart rate at the 1st minute after the end of the 3MISP test.

**Table 3:** Multiple regression models for predicting VO_2max_ using independent values in the training group.

VO_2max_ (mL·kg^−1^·min^−1^)	3MISP_30s_ Model			3MISP_60s_ Model		
	Unstandardized Coefficients	Standardized Coefficients	*p*	Unstandardized Coefficients	Standardized Coefficients	*p*
Constant	47.534		<0.001	49.357		<0.001
Age (years)	−0.131	−0.182	0.001	−0.143	−0.199	<0.001
Gender (women = 0, men = 1)	2.506	0.182	0.003	3.084	0.224	<0.001
PBF (%)	−0.361	−0.353	<0.001	−0.348	−0.340	<0.001
HR0 (bpm)	−0.085	−0.139	0.033	−0.107	−0.173	0.007
∆HR3−HR4 (bpm)	0.260	0.318	<0.001	0.259	0.290	<0.001
*F*	48.571			48.401		
*P*	<0.001			<0.001		
R^2^	0.644			0.644		
Adjusted R^2^	0.631			0.630		
SEE (mL·kg^−1^·min^−1^)	4.2043			4.2090		
SEE%	12.283			12.297		
R^2^*_p_*	0.646			0.651		
SEE*_p_*	4.180			4.134		

PBF, percent body fat; HR0, heart rate at the beginning of the 3MISP test; ΔHR3−HR4, the difference between the heart rate at the 3rd minute during the exercise and the heart rate at the 1st minute after the end of the 3MISP test; R^2^*_p_*, PRESS squared multiple correlation coefficient; SEE, standard error of estimate; SEE%, SEE/mean of measured VO_2max_ × 100; and SEE*_p_*, PRESS SEE.
